# Prevalence of peripheral arterial disease in patients with heart failure with preserved ejection fraction

**DOI:** 10.6061/clinics/2019/e978

**Published:** 2019-10-09

**Authors:** Giuliano Reolon da Cunha, Roberto José Brugnarotto, Victória Armendaris El Halal, Márcio Garcia Menezes, Eduardo Bartholomay, Luciano Cabral Albuquerque, Luiz Cláudio Danzmann

**Affiliations:** Cardiologia, Universidade Luterana do Brasil, Canoas, RS, BR.

**Keywords:** Ankle-Brachial Index, Heart Failure, Peripheral Arterial Disease

## Abstract

**OBJECTIVES:**

To describe the prevalence of the reduced ankle-brachial index (ABI) in patients with heart failure (HF) with preserved ejection fraction (HFpEF) attended at a HF clinic in the metropolitan region of Porto Alegre, and to compar the patients to those with reduced ejection fraction (HFrEF).

**METHODS:**

A descriptive observational study, included patients referred to the heart failure clinic in HU-Ulbra with HFpEF or HFrEF and diastolic dysfunction, and measurements of ABIs using vascular Doppler equipment were performed in both groups.

**RESULTS:**

The sample consisted of 106 patients with HF, 53.9% of the patients had HFpEF, and 19.4% had a diagnosis of peripheral arterial disease (PAD) (ABI less than 0.9). PAD was identified in 24.1% of the patients with HFpEF, while15.8% of patients in the HFrEF group were diagnosed with PAD.

**CONCLUSION:**

Our results did not identify a significantly different prevalence of altered and compatible PAD values in patients with HFpEF. However, we showed a prevalence of 19.4%, a high value if we consider similar populations.

## INTRODUCTION

Heart failure (HF) is a prevalent clinical syndrome in the world population, and approximately two-thirds of cases present coronary artery disease (CAD) as the main etiology (1), with more than 50% of cases in North and Europe and 30% to 40% in Asia, Latin America and the Caribbean (2); HF is associated with a more reserved prognosis after acute myocardial infarction (AMI) (3).

Peripheral arterial disease (PAD) is also associated with high mortality rates and cardiovascular events and affects approximately 200 million people worldwide (4). Both HF and PAD present common risk factors, such as diabetes mellitus, systemic arterial hypertension (SAH), obesity, increased age, smoking, inflammation, and atherosclerosis (5). This condition increases the cardiovascular risk for the onset of HF and is an independent factor of hospitalization and mortality (6).

Measurement of the ankle-brachial index (ABI) has already been established in clinical practice for the diagnosis of PAD (7-9). Studies correlate a low ABI (≤0.9) with a higher incidence of cerebrovascular diseases, specifically in patients with HF (7). However, PAD is underdiagnosed and/or undertreated, which makes it a complication factor (3,10).

Considering this rationale, the present study aims to describe the prevalence of PAD, with a diagnosis made through ABI measurement, in patients with HF with preserved ejection fraction (HFpEF) attended at a HF clinic of a nontransplant hospital in the metropolitan region of Porto Alegre.

## METHODS

The study population was composed of adult patients over 18 years of age who were followed up at the Heart Failure Outpatient Clinic of the University Hospital (HU) of the Lutheran University of Brazil and were consecutively referred for diagnosis of HF according to the Boston criteria from the general cardiology outpatient clinic of the same institution. Patients with musculoskeletal alterations or ulcers in the lower limbs that obstructed access to the brachial, posterior tibial or pedal arteries and consequently, the measurement of ABI or those who refused to participate in the study were excluded.

The data were collected according to a protocol approved by the research ethics committee - CEP of the ULBRA (358/2010), and all the patients who fulfilled the inclusion criteria signed a free and informed consent form, according to the annex. The collection of data and records was carried out by volunteer researchers responsible for the referral of the patients to the HU heart failure outpatient clinic. Demographic, clinical, echocardiographic, and ABI values were recorded. The diagnosis of HFpEF followed the current European Society of Cardiology criteria (11), which include HF signs and symptoms associated with diastolic dysfunction criteria and LV ejection fraction greater than or equal to 50%, both defined by transthoracic echocardiography (11).

The ABI measurement was performed by two previously trained researchers using vascular Doppler equipment (MARTEC, model DV-600, São Paulo, Brazil) and an aneroid sphygmomanometer (Premium) duly calibrated for the estimation of systolic pressure of the ankle, using the posterior or pediatric tibial pulse and brachial systolic pressure and using the right and left brachial pulse. The technique to perform ABI was based on the guidelines of the European Society of Cardiology (12) and was used to determine the ratio between the higher ankle/pedal blood pressure (SBP) and the respective systolic brachial pressure (SBP) measured on both sides. The range of normality is between 1.00 - 1.39, with values of 0.91 to 0.99 considered the lower limit and 1.3 to 1.4 the upper limit. Values less than or equal to 0.9 were considered criteria for the diagnosis of PAD; those above 1.4 are related to arterial stiffening (12).

### Statistical Analysis and Data Processing

The frequency data are presented as the means, standard deviations and percentages. Continuous variables are presented as the means and standard deviation and compared using a paired Student's t-test for independent samples in the comparison between groups. Categorical variables are expressed as percentages and compared by the Chi-square test. P values less than 0.05 were considered indicative of significance. Statistical analysis was performed using IBM SPSS software version 23.0 (Statistical Package for Social Sciences, USA)

## RESULTS

A total of 112 patients were included, and it was possible to measure ABI in 106 of them; 6 were excluded because of one of the exclusion criteria: one due to the presence of venous ulcers, two due to refusal to perform the measurements and amputation of the lower limb, and two because of incalculable measures of ITB due to advanced disease.

The mean age was 65.7±11.8 years, the mean BMI was 29.3 kg/m^2^, and 53.4% of the participants were female. Class II of the New York Heart Association functional classification prevailed in our sample (Table 1).

The prevalence of ischemic heart disease as a basic pathophysiology in the development of HF was 68% (Table 1).

The population presented a prevalence of 53.9% of the diagnosis of HFpEF (Table 1). The prevalence of ABI in the general population compatible with a diagnosis of PAD was 19.4% and was not significantly different between patients with HFpEF and heart failure with reduced ejection fraction (HFrEF) (24.1% *versus* 15.8%, *p*=0.442), respectively. The prevalence of smoking was higher in the HFpEF group (Table 2).

## DISCUSSION

The population in the present study shows a high prevalence of patients with HFpEF. This observation may be related to the bias of the institution to have an IC clinic dedicated to the assistance and research of this clinical syndrome. In this context, the general characteristics of our HFpEF sample are similar to those described in most publications, and our sample has a higher proportion of women and more advanced age, as is the case with current registries (13). In addition, we observed in our population that smoking, a factor typically associated with atherosclerosis and PAD, was significantly higher in patients with HFpEF.

The prevalence of the diagnosis of PAD by ABI in the general population was high (19.4%), but the difference was not statistically significant when the population was dichotomized by the ejection fraction of the left ventricle in the HFpEF and HFrEF (24.1%x15.8%, *p*=NS). Several factors may explain this phenomenon, including atherosclerotic load and the distribution of ischemic heart disease rates (14).

In two meta-analyses, Hajibandeh S, et al. (15) and Hao Z, et al. (16) assessed a high risk of CAD (outcomes composed of AMI) in patients with reduced ITB. Likewise, a study by Hisayama (17) found a 4.11-fold risk of CAD in patients with reduced ABI without previous CVD. Moussa I, et al. (18) observed a PAD prevalence of 15% in patients diagnosed with CAD, a rate that was also similar to our data. However, in our data, similar rates of CAD were registered in the groups with HFrEF and HFpEF (55.6%x44.4%, *p*=NS), which may have balanced the atherosclerotic loads and the chances of ABI indicative of PAD.

Evaluating the phenomenon from another perspective, Khaira KB, et al. (19) investigated the prevalence of HF in patients with PAD at more advanced stages. Of the 381 patients in their study, 31% had a previous history of HF, and the majority of the patients (62%) had HFpEF. At the same time, we had a high rate of HFpEF (53.9%), and the affected individuals had a high rate of PAD (24.1%). As already demonstrated in the MAGGIC study (20), this result suggests that the profile of patients with HFpEF, with more advanced age and a higher number of comorbidities, would facilitate a presentation of a more chronic, slower-growing and mature atherosclerosis load related to the peripheral arterial vessel. This association of HFpEF and PAD can still be considered a plausible hypothesis to explain the peripheral component of heart failure in this population, that is, muscle insufficiency in response to an increase in demand could be explained, at least in part, by arterial insufficiency in approximately one-quarter of our patients (20).

A risk factor for atherosclerotic disease that was significantly different between the two HF groups was smoking, being more prevalent in the HFpEF population (26%x16%, *p*=0.001). This factor is cited as associated with the risk of PAD in several studies and may have contributed to the high rates of diagnosis of PAD in both populations, although mainly in patients with HFpEF (21,22).

However, when we compare our results with other studies of populations with HF, we are surprised by our rates of PAD. A post hoc analysis of 2331 subjects included in the randomized controlled trial (HF-ACTION) (23) testing physical training for HF patients found a PAD rate of 6.8%. Likewise, another study analyzing 28,771 patients with LV dysfunction or HF after AMI, combined in a meta-analysis of four randomized trials, observed a prevalence of only 8.2% of PAD (6). In both studies, the prevalence of PAD was much lower than ours, leading to the hypothesis that our population is actually more severe or that the disease criteria used are more sensitive than the cited publications.

Our results corroborate the current recommendations for the European Society of Cardiology and European Society of Vascular Surgery (12), which emphasize the importance of establishing this diagnosis due to the independent predictive power for hospitalization and all-cause mortality in patients with HF (4,6,9,15,16,24) and a risk factor for cardiovascular events, except stroke, in those without a previous history of HF (6,19).

### Limitations

Our study has limitations regarding the number of participants involved, because we analyzed all patients with data available in our database and may demonstrate data that are more consistent with the natural increase in the study sample in the future. In addition, the potential for ITB calibration bias should always be considered due to its inter- and intraobserver variability.

## CONCLUSION

Our results identified a high prevalence of PAD diagnosed by arterial Doppler-mediated ABI in patients diagnosed with HFpEF; however, there was no statistically significant difference in these rates compared to patients with HFrEF. These results point to a trend and deserve to be further investigated in the future.

## AUTHOR CONTRIBUTIONS

Cunha GR performed the data collection, project design and research, updated and revised the patients’ general database, and assisted in statistical analysis. Brugnarotto RJ performed the data collection and updated the patients’ database. El Halal VA updated the patients’ database. Menezes MG was responsible for the statistical analysis, updated the general database and assisted in structuring the project. Bartholomay E and Albuquerque LC provided active and essential contribution in structuring and revising the project and manuscript. Danzmann LC guided the whole project and was responsible for the design, research and structure of the manuscript, updated the general database of patients, reviewed the data and assisted in statistical analysis.

## Figures and Tables

**Table 1:** Clinical Characteristics of Patients with ICFER and ICFEP.

N=106 patients	
Variables	Values
**Anthropometric Data**	
Age (years)	65.75±11.88
BMI (kg/m^2^)	29.38±7.14
Female (%)	53. 4
CFNYA I (%)	30.3
CFNYA II (%)	44.8
CFNYA III (%)	20
CFNYA IV (%)	3.6
**Comorbidities (%)**	
Arterial hypertension	83.2
Diabetes mellitus	39.5
Dyslipidemia	54.7
AMI	30.5
Arrhythmia	16.8
FHSD	13.7
Smokers (%)	14
Ex-smokers (%)	47
Nonsmokers (%)	39
**Medications (%)**	
Beta Blocker	74.7
ACE	41.6
ARB	40.3
BCC	35.2
Furosemide	52.1
Thiazide	*31.6*
Spironolactone	52.1
**Variables ABI and ECO**	
ABI changed (%)	19.4
ABI minor	0.79±0.16
ABI greater	1.11±0.12
LVEFr (%)	46.1
LVEFp (%)	53.9
	

BMI: body mass index; CFNY: functional class of the New York Heart Association; ACE inhibitors: angiotensin converting inhibitor; ARB: angiotensin receptor blocker; BCC: calcium channel blockers; AMI: acute myocardial infarction; FHSD: family history of sudden death; ABI: ankle-brachial index; LVEFr: left ventricular ejection fraction reduced; LVEFp: left ventricular ejection fraction preserved.

**Table 2:** Comparison between the HFpEF and HFrEF groups.

Variables	HFpEF	HFrEF	*p*
**ANTHROPOMETRIC DATA**			
Age (years)	66.84±10.88	63.97±12.87	0.028
Weight (kg)	80.09±17.05	79.80±17.07	0.615
Stature (cm)	1.62±0.93	1.66±0.94	0.884
Sex (%)	67.4	36.4	0.001
**COMORBIDITIES**			
Dyslipidemia (%)	64	60.5	0.653
Diabetes mellitus (%)	40.7	48.4	0.307
Arterial hypertension (%)	94.2	88.4	0.173
AMI (%)	29.8	40.8	0.098
Stroke (%)	1.7	3.3	0.506
Smokers (%)	26%	16%	0.001
**CLINICAL DATA**			
LVEF Teicholz	67.34±8.52	34.32±8.29	0.001
ABI >0.90	1.11±0.11	1.13±0.13	0.531
ABI ≤0.90	0.71±0.18	0.75±0.11	0.617
ABI ≤0.90 (%)	24.1	15.8	0.442
NYHA I (%)	30.6	31.2	0.650
NYHA II (%)	48.2	40.8	0.503
NYHA III (%)	18.8	22.5	0.641
NYHA IV (%)	2.4	5.6	0.703
FHSD	18.1	13.2	0.514
**Etiology**			
Ischemic (%)	55.6	44.4	0.468
**MEDICATIONS**			
Beta blocker (%)	70.5	90.9	0.001
ACE (%)	39.8	50.6	0.209
ARB (%)	44.3	39	0.486
Furosemide (%)	37.5	76.6	0.001
Thiazide (%)	54.5	11.7	0.001
Spironolactone (%)	40.9	71.4	0.001

HFpEF: Heart failure with preserved ejection fraction; HFrEF: Heart failure with reduced ejection fraction; AMI: acute myocardial infarction; Stroke: stroke; LVEF: left ventricular ejection fraction; ABI: ankle-brachial index; CFNY: functional class of the New York Heart Association; FHSD: family history of sudden death ACE inhibitor: angiotensin converting enzyme inhibitor; ARB: angiotensin receptor blocker.
